# On the Functions of the Nervous System

**Published:** 1815-02

**Authors:** C. W. Smerdon

**Affiliations:** Member of the Royal College of Surgeons.


*For the Medical and Physical Journal.*

### *On the Functions of the Nervous System;* by C. W. Smerdon, Member of the Royal College of Surgeons.

THE Priestleyan theory on the effects of Respiration, is at once so beautifully simple, and (considering the animal machine as mere congeries of chemical apparatusses) so readily accounts for the phenomenon of animal heat, as well as the change which the blood undergoes in the lungs, that we cannot wonder why the human mind should have become dazzled with its splendour, and consequently as much wedded to it by prejudice, as by the conviction of its being built on the immutable basis of truth; and hence it is, that, notwithstanding this beautiful fabric is raised upon a very sandy foundation, it is still generally esteemed as truth, and will always remain a splendid monument of human genius.

We are taught, by this doctrine, that the blood in the pulmonary pulmonary arteries is charged with carbon and hydrogen in loose combination; that these, being attracted by oxygen in the bronchial tubes and cells, pass into them (probably) by the exhalents, where one portion of the oxygen, uniting with the hydrogen, forms water, while another portion changes the carbon into carbonic acid: the blood, being thus freed from its impurities, is rendered capable of containing an additional quantity of caloric in a latent form, and this heat it acquires from that which is let loose during the process I have mentioned.

From the great bulk of the pulmonary arteries, when compared with the corresponding veins, we might be certain that something is evacuated from their contents, even if we were ignorant of the moisture which is expelled with the air in expiration, but that this something is hydrogen, is, I think, at best very doubtful; we know the exhalents of the lungs bear a very close resemblance, both in their structure and ultimate intention, to the perspiratory organs of the skin, which, in their healthy state, are constantly pouring out a fluid, and similar sets of vessels are also to be found in all the cavities of the body; in the abdomen, for instance, the contiguous arteries, after ramifying minutely over the peritoneum, terminate by open mouths or pores, from which serum, in the form of vapour, is exhaled, for the purpose of lubricating the contained viscera. The exhalations from the lungs, however, arise for the ramification of arteries containing venous blood, and, in common with the perspiration from the folicular openings of the skin, is an excretion evidently intended to evacuate the more watery part of the blood.

It is utterly impossible for the mere chemist, unless he call to his aid the science of physiology, to account for the apparent changes which take place in the temperature of man, when under the influence of some morbid disposition. If animal heat be first generated in the lungs, if it be imbibed by the blood, and again set free in the course of circulation, the body, under every circumstance, as well in health as in disease, ought to be equably heated: how is it then that such alternations take place in the temperature of the body as we witness in fever? how is it that these alternations come on after regular intervals, and in regular successions? how can we account for the accumulation of heat in one part of the body, while every other part is perfectly healthy, which is the case in local inflammations? The cause of this disease is said to be an accumulation of blood in the vessels of the inflamed part: if this be true, and according to the Priestleyan doctrine, the part, instead of being sensibly hotter, ought to be colder than usual; at least there should be no increase of temperature, because the circulation in it being rather retarded in consequence of debility in the extreme branches, fresh blood cannot so readily supply the place of that which has parted with a portion of its heat.*

The phenomenon of that sort of local inflammation which is dependant on a constitutional cause, is equally inexplicable. Of this, gout is a striking example. A person, for instance, who has gone to bed in apparent health and vigour, shall be awoke in the night with excruciating pain in the ball of his great toe, accompanied with great increase of temperature, tumefaction, and redness of the part, and symptomatic fever; after the gouty paroxysm has continued for a definite period, it gradually subsides, leaving the sufferer in comparative health. Now, according to the generally-received opinion of animal heat, whence comes this increase of temperature, which, together with pain, generally precedes the swelling and redness? does it proceed from a greater quantity of blood than usual circulating through the affected part? This is not probable, because, after the paroxysm subsides, notwithstanding the pain and heat are considerably lessened, the part remains as red as before, which could not be the case if the increase of temperature arose from an increased quantity of blood; at least it is but fair to infer, that, if the cause remain unabated, the effect would also continue. After the intermission has remained for a certain time, during which the person has continued comparatively well, he will have another paroxysm of gout resembling the first in its train of symptoms, its duration, and its gradual disappearance.

The symptoms of every sort of unhealthy local inflammation are exasperated towards evening. A chilblain is an instance of this species of disease: here the pain and burning heat of the part during the exacerbation amount almost to torture; still the distress, after a few hours, will gradually subside, leaving the part swollen and of a purple redness, sore indeed to the touch, but free from pain, and of the natural degree of heat.

However satisfactorily, then, the Priestleyan theory may explain the cause of animal heat, when the functions of the

---

* Dr. T. Thompson, in his Annals of Philosophy, informs us, that in the course of four days a small inguinal gland gave out a quantity of heat sufficient to have raised the temperature of seven pints of water from 40° to 212°; yet the temperature of the part, at the end of the experiment, was not less than that of the rest of the body. The inflammation was subdued.

body body are carried on in a uniform manner, it is inadequate to account for the variations in the temperature of man, when these functions are deranged by disease; and, as it is but reasonable to suppose that these phenomena proceed from one common cause, which, when healthy, produce equable effects, and *vice versâ,* so it cannot be considered as presumptuous or unreasonable to reject a doctrine which is evidently erroneous.

The experiments of Mr. Brodie, while they throw a considerable degree of light on this important subject, inflict a death blow on the Priestleyan doctrine. This gentleman found, that, after the apparent death of an animal was produced by pithing or decapitation, respiration could be artificially kept up for a definite period, as well as the diastole and systole of the heart; and, what is still more to our purpose, this organ still continued to circulate arterial and venous blood in the same manner (but, perhaps, not in such quantities) as when the animal was really alive. Still, notwithstanding this demonstrative proof, that the same processes were going on in the lungs, which, we are told, necessarily converts a quantity of latent into free caloric, the animal cooled faster than another placed under similar circumstances, but in which respiration was not kept up.

These experiments* (if accurately conducted) render it very probable that the brain is absolutely necessary for the production or distribution of animal heat.

Mr. Hunter suspected that the nervous system, if not the

---

* Whoever reads the detail of these ingenious experiments, must feel as convinced as I do of their accuracy; and still this has been called in question very lately by similar experiments instituted by a foreigner, from which almost opposite results were produced. Now, even if these experiments be correctly stated, it would be extremely unjust to infer that they directly prove an inaccuracy in those of Mr. Brodie. None of the animals were decapitated, and the gentleman (I do not recollect his name) tells us, with an air of some importance, that, in destroying them, he did not cut through any blood-vessel. Now, if he divided the spinal marrow between the atlas and foramen magnum, he must have been fortunate indeed not to wound the vertebral arteries; and if he divided it lower in the neck, allowing the par vagum on each side also to be cut through, (for, if I am correct, a dog has no cervical sympathetic nerves,) would not some nervous communication be kept up between the brain and the trunk by means of the nervi accessorii? Other nerves also might be mentioned which I strongly suspect would contribute to that purpose.

source, source, was a very principal agent, of animal heat; and he was led to adopt this opinion by observing considerable alternations of temperature in a gentleman during a paroxysm of apoplexy.

The derangement which fever occasions in the functions of the brain, as well as in the temperature of the body, is particularly striking. In the cold stage of an intermittent, the nervous system evidently becomes enfeebled, the mind dull and confused, and the patient a poor shivering being, sinking, as it were, voluntarily into the grave. After symptoms of diminished energy have continued for some time, the progress of the disease is suddenly arrested by an unknown agent; the mind now becomes quick and irritable, and its ideas rapid even to delirium, while the heat of the body gradually returns, and at length exceeds the natural degree.

If we could identify, by a direct experiment, the connection which appears to exist between the matter of electricity or galvanism and nervous energy, we could not be at any loss to demonstrate the cause or causes of animal heat, because the functions of the nerves, and the generation of caloric, would follow in the relation of cause and effect. If, however, this be not in our power, on account of the utter impossibility of explaining the modus operandi of powerful agents which we cannot bring under the cognizance of our senses, the evidence which we derive from comparing the effects of these matters on animals, prove this similarity in a very striking manner. The effects of galvanism on a recently-dead animal are too well known for me to describe them. On the living subject, also, the effects of this peculiar stimulus, when applied to the organs of the senses, are not less remarkable; and it would seem that galvanism has the property of heightening their several powers. Writers on this subject inform us, that, if the wires proceeding from a galvanic battery be applied to a point above the eye-lids, a flash of vibratory white light will be produced, in a dark room; if one of the wires, previously moistened, be placed in the ear, while the other is held by the hand in a glass of water, a stroke and a strong sound will be perceived in the ear; if the wire from the zinc end of the battery be placed on the tongue, and the conductor from the copper end under the tongue, a very disagreeable pungent taste will be perceived. These remarkable phenomena are strong presumptive evidence in favour of an analogy between the effects of galvanism and nervous fluid: each pass through conductors with the most astonishing celerity, and, although they are the most powerful agents in nature, their state of existence is perfectly unknown. The conductors of the nervous fluid are also good conductors of the galvanic; and we know that the effects of both are similar, when applied through the medium of the nerves to the moving fibres of the body; and, lastly, I will repeat, that the powers of the senses are heightened when under the influence of galvanism. Hence, it is evident, that if the matter emanating from the brain, and that which proceeds from a galvanic battery, be not the same, the effects proceeding from their actions on the structure of animals are strikingly similar; nor is it unreasonable to conclude, that, if the agency of nervous energy could be brought into action out of the body, the same phenomena would proceed from it as we know are produced by galvanism.*

The nerves and arteries, even to their ultimate branches, uniformly accompany each other. No part of the body, for instance, can be pricked, without producing pain, or drawing blood, while the bones, which are but scantily supplied with that fluid, are almost insensible. In the liver, each branch of the vena portarum has its accompanying nervous filament. In the lungs, also, the ramifications of the bronchial and pulmonary vessels are accompanied by branches of the pulmonic plexus; nor is this equality between the arteries and nerves to be seen only in the larger organs of the body, but it is highly probable that wherever a fluid escapes from the extreme branch of an artery, whether it be a secretion, properly so called, or merely a percolation from the vessel, there also a nervous filament will be found.

This remarkable uniformity in the distribution of the nervous and arterial symptoms would lead one to suspect that there is a reciprocity of action between them, without which their ultimate intentions for sensation on the one hand, and for nourishment and secretion on the other, could not be accomplished.

---

* It is, perhaps, necessary for me to state, that these observations were drawn up (rather, indeed, for my own amusement, than with any ulterior object) before I had seen the elegant Lectures of Mr. Abernethy on the "Rationality of Mr. Hunter's Theory of Life;" and it is under the shelter of opinions which he has therein so ably defended, that I have presumed to disseminate my ideas on a subject which, in a physiological point of view, is of so much importance. Perhaps, also, I have been prompted by some degree of national pride, since the cause of animal heat has been very lately ascribed to fermentation, by a member of the most learned body in France.

If

If the analogy between the actions of the brain and of a galvanic battery be established, it will open to us a wide field for the exercise of our reasoning faculties, and, besides giving us an insight into the modus operandi of the secretory and excretory organs, it will furnish us with a rational explanation of the causes of these phenomena, which are the subject of this paper.

To the chemist, the agency of galvanism is the most powerful of any that he is acquainted with: its influence in the decomposition of various substances which were formerly considered as elementary, is too well known for me to particularize, nor is it my intention to discuss a subject which is foreign to my purpose; it is sufficient for me to say, that this peculiar stimulus is the most powerful agent for destroying that property which cements the integrant particles of bodies together.

If, then, the matter which emanates from the brain and spinal marrow, and conveyed to the different parts of the body through the medium of nerves, be analogous, either in its properties or its actions, to galvanic fluid, what will be the probable effect of that matter when it comes in immediate contact with blood contained in the extreme branch of an artery, or in that part where the vessel runs into a vein, after giving off an excretory duct or an exhalent pore? Is it not reasonable to suppose that this blood will become decomposed, partially or completely, in proportion to the quantity or energy of the nervous fluid which is sent to it? and knowing, as we do, that, at the instant a body is decomposed by a stream of galvanic fluid, a spark is produced, is it not evident, that if there be such an action constantly going on between the arterial and nervous branches in every part of the body, that the generation of heat must be the natural effect of the process? and lastly, may we not conclude, that, at this instant, a portion of blood is burnt or carbonised?

These phenomena (which, I presume, are evidently what must follow as the natural effect of actions that are analogous to galvanism) will be still farther illustrated when we come to the consideration of the secretion and excretion, and which will form the subject of my next communication.

*Clifton, Bristol;* C. W. SMERDON.

